# Effects of paternal age and interaction with smoking, alcohol consumption and maternal age on implantation failure in women undergoing ART

**DOI:** 10.1038/s41598-025-03651-y

**Published:** 2025-07-02

**Authors:** Jiaqian Yin, Ruoling Chen, David Churchill, Huijuan Zou, Peipei Guo, Chunmei Liang, Xiaoqing Peng, Jieyu Wang, Zhikang Zhang, Weiju Zhou, Yunxia Cao

**Affiliations:** 1 Faculty of Education, Health and Wellbeing, University of Wolverhampton, Wolverhampton, WV1 1DT UK; 2 New Cross Hospital, Wolverhampton, Wolverhampton, UK; 3 Department of Obstetrics and Gynecology, the First Affiliated Hospital of Anhui Medical University, No 218 Jixi Road, Hefei, 230022 Anhui China; 4 NHC Key Laboratory of Study on Abnormal Gametes and Reproductive Tract (Anhui Medical University), No 81 Meishan Road, Hefei, 230032 Anhui China; 5 Biopreservation and Artificial Organs, Anhui Provincial Engineering Research Center, Anhui Medical University, No 81 Meishan Road, Hefei, 230032 Anhui China; 6 Vanke School of Public Health, Tsinghua University, Beijing, China

**Keywords:** Advanced paternal age, Implantation failure, Infertility, In vitro frozen embryo transfer, Assisted reproductive technology, Reproductive disorders, Lifestyle modification, Risk factors, Epidemiology

## Abstract

It remains unclear whether there is an independent association of paternal age with implantation failure (IF) in couples undergoing Assisted Reproductive Technology (ART) treatment. Little is known about the interactive effects of paternal age with maternal age, paternal smoking and alcohol consumption on IF. The Anhui Maternal-Child Health Cohort Study (AMCHS) interviewed 1910 infertile couples (paternal age ranged 22–57 years) to look for risk factors associated with IF. Implantation outcome was assessed using serum β-HCG between 0 and 25 IU/L 14 days post-embryo transfer. Odds ratios (ORs) for IF in relation to paternal age, and its interactions with maternal age, paternal smoking and alcohol consumption were evaluated using logistic regression models. The risk of IF (*n* = 735) increased with paternal age ≥ 35, while it remained similarly lower among males aged 22–34. After adjusting for socioeconomic status,  paternal lifestyles and semen factors, the OR for IF was 1.50 (95%CI 1.16–1.93) at paternal age of 35-<40 years, 2.06 (1.34–3.16) at 40-<45 years and 3.80 (2.50–7.07) at ≥ 45 years in comparison to those aged < 35. With additional adjustments for maternal age and maternal factors including anovulation and antral follicle counting, the corresponding ORs were 1.19 (0.89–1.60), 1.31 (0.79–2.16) and 2.13 (1.06–4.29). The association remained significant in those with maternal age > 30, paternal smoking or alcohol consumption, but not in their counterparts, respectively. Among those with a paternal age ≥ 35, each additional year of smoking was associated with a 7.0% increased risk of IF (OR = 1.07, 95% CI: 1.01–1.14); however, no such association was observed in younger paternal age groups. Significant interaction effects were observed between advanced paternal age and maternal age, as well as paternal alcohol consumption. The AMCHS had demonstrated that paternal age ≥ 35 years elevates the risk of IF, with additional impacts from advanced maternal age and paternal alcohol consumption. The findings of the AMCHS highlight the need to consider paternal and maternal ages in fertility planning and suggest that IF can be reduced by stopping paternal smoking and alcohol consumption.

**Supplementary Information:**

The online version contains supplementary material available at 10.1038/s41598-025-03651-y.

## Introduction

Infertility affects more than 14% of couples worldwide, with male factors accounting for approximately 30% of cases. As infertility rates rise, the demand for Assisted Reproductive Technology (ART) has been increasing^1,2^. However, the chance of conception per cycle remains relatively low and about 35% of euploid embryos suffer implantation failure (IF)^3^ which refers to the inability of a zona-free blastocyst to attach and subsequently penetrate the uterine lining^4^.

While advanced maternal age is a well-documented risk factor for IF^5,6^, the influence of paternal age on the outcome of ART remains unclear. Some studies^7,8^ have shown an inverse association between paternal age and IF, but others have not^9,10^. The reason for these inconsistent results remains unknown. They might stem from varied adjustments undertaken during data analysis or a specific paternal age threshold associated with increased risk of IF. Furthermore, although a significant positive correlation between paternal and maternal ages exists^11^, few studies have assessed to what extent maternal age interacts with and explains the association between advanced paternal age and risk of IF. In Japan, Kidera et al.^12^ showed that advanced paternal age decreased the normal fertilization rate when the maternal age was 35–39 and increased the miscarriage rate when maternal age was < 35 years, suggesting advanced parental age may jointly increase the risk of IF. The potential mediating role of maternal age in the relationship between advanced paternal age and IF remains to be quantified.

Previous studies have suggested associations of paternal smoking and alcohol consumption with an increased risk of IF^13–16^. However, the interaction between advanced paternal age, smoking and alcohol consumption on the increased of IF is unclear. It has been hypothesised that advanced paternal age, smoking and alcohol consumption could jointly increase oxidative stress and DNA damage in sperm^15^. In this paper, we carried out a prospective cohort study of women undergoing their first frozen embryo transfer (FET) in China^17^. The study aimed to elucidate if there was an independent association between paternal age and IF. Additionally, we assessed the interaction effects of paternal age with maternal age, paternal smoking, and alcohol consumption on the risk of IF.

## Methods

### Study population and data collection

In 2017, the Anhui Maternal-Child Health Cohort Study (AMCHS) was set up in Anhui China, to investigate the ART outcomes and their influencing factors. The AMCHS methods have been fully described in our previous paper^17^. In brief, the AMCHS enrolled 2,198 infertile couples consecutively, undergoing FET for the first time treatment with autologous gametes from May 2017 to April 2021 at a large university-affiliated infertility center in China. In this study, the focus was FET, therefore couples having preimplantation genetic diagnosis technology (PGT) and fresh embryo transfers were not included. Informed consent was obtained from each participant. The primary interview instrument was a comprehensive questionnaire addressing general health and risk factors, details of which have been previously published^17^.

This study was approved by the Ethics Committee of Anhui Medical University (No. 20160270) and complied with the Helsinki Declaration.

### Ovarian stimulation protocol, embryo quality assessment and frozen embryo transfer protocol

Controlled ovarian stimulation was achieved using a standard long pituitary downregulation in the luteal phase or a short protocol with an antagonist to treat infertility. In the AMCHS, ejaculated spermatozoa were used. Semen was separated through pure sperm density gradient or swim-up sperm preparation. All semen samples were analysed according to the World Health Organization criteria^18^. Either standard IVF or Intracytoplasmic Sperm Injection (ICSI) was employed to fertilize oocytes in the fertilization medium. A good-quality blastocyst was defined as B3–B4 or B5 embryo ≥ BB based on the Gardner standard^19^. Two or fewer embryos were transferred depending on their quality on day 5 or 6 after oocyte retrieval in compliance with national regulations. All participants had a serum hCG level done on day 14 after embryos transfer and an ultrasound scan examination was undertaken on thirty days after embryos transfer to confirm the presence of a fetal sac.

### Outcome measurement

Implantation failure was defined as, a serum β-HCG (Beckman Coulter Inc., Fullerton, CA, USA) below 25 IU/L on 14 days post-embryo transfer and the absence of a fetal sac at the ultrasound examination.

### Statistics analysis

The analysis included data from 1,910 couples after excluding those with missing paternal age (*n* = 6), withdrawals (*n* = 78), blastocyst cultivation failures (*n* = 28), low responses to controlled ovarian hyperstimulation (*n* = 19), natural pregnancies occurring mid-treatment (*n* = 5), and unavailable outcome data (*n* = 152). Their characteristics were described using means ± SD for continuous variables and numbers and % for categorical variables. Differences between the two groups, implantation success and implantation failure, were examined using t-tests for continuous variables and Chi-square tests for categorical variables. Binary logistic regression models were employed to calculate odds ratios (ORs) and 95% confidence intervals (CIs) for the implantation failure in relation to paternal age. Using logistic regression analysis, we adjusted for different sets of confounders to examine the effect sizes of different factors from the paternal and maternal sides, on the association of advanced paternal age with IF. In model 1, we adjusted for socioeconomic status (residential location and family income), paternal smoking habits and alcohol consumption. In model 2, we further adjusted for paternal mental status (depression and anxiety), histories of any serious illness, and semen volume and morphology. In model 3, maternal age was additionally adjusted to examine how great the residual effect of maternal age could have on the association of advanced paternal age with IF. Finally, in model 4, we included other factors from females as adjustments, including female lifestyles, body mass index (BMI), mental status, infertility type, infertility duration, ovulation disorder and antral follicle count.

In addition, we investigated the interaction effects of paternal age with maternal age, paternal smoking and alcohol consumption respectively, using the terms of advanced paternal age, advanced maternal age, smoking, or alcohol consumption in the multiple adjusted regression models. We also stratified the data analysis by advanced maternal age (or paternal smoking and alcohol consumption, respectively) to examine the differences in the association of advanced paternal age with IF (additive effect) between two groups using the methods in our previous studies^20^. All analyses were performed using SPSS version 29.0 (IBM Co., Armonk, NY, USA).

## Results

Table 1 shows the characteristics of male participants. Of the 1910 male participants, their average age was 32.3 years (SD 5.1), with a range of 22–57 years. 40.3% had an educational level of ≥ university, 51.9% were current- or former- smokers and 71.3% consumed alcohol in different amounts. Compared to those with implantation success, men who had implantation failure were more likely to be older and living in urban areas (Table 1). There were no significant differences between the two groups for implantation success and failure in the other characteristics (Table 1). However, the data of their spouses showed that women with implantation failure were more likely to be older, suffer from anxiety, have secondary infertility, higher oestradiol levels, < 10 antral follicles count and were less likely to be anovulatory (data on request). As expected there was a significant correlation between paternal age and maternal age (*r* = 0.789, *p* < 0.001).

**Table 1:** Characteristics of male participants: AMCHS, China.

Variable	All participants	Implantation success	Implantation failure	P^†^
	*N* = 1910 (%)	*n* = 1175 (%)	*n* = 735(%)	
Age (mean, SD)	32.32 (± 5.06)	31.75 ± 4.63	33.23 (± 5.58)	< 0.001
Socioeconomic status				
District				
Urban	1459 (76.4)	875 (74.5)	584 (79.5)	0.014
Rural	449 (23.5)	299 (25.4	151 (20.5)	
*Missing data*	*2 (0.1)*	*1 (0.1)*	*1 (0.1)*	
Educational level				
University MSc or PhD	175 (9.2)	113 (9.6)	62 (8.4)	0.679
University first degree	594 (31.1)	362 (30.8)	232 (31.6)	
3-year Diploma	491 (25.7)	288 (24.5)	204 (27.6)	
High School/ Secondary technical School	333 (17.4)	211 (18.0)	122 (16.6)	
Junior high school	299 (15.7)	191 (16.3)	108 (14.7)	
Primary school or below	13 (0.7)	7 (0.6)	6 (0.8)	
*Missing data*	5 (0.3)	3 (0.3)	2 (0.3)	
Occupation				
Officers	510 (26.7)	305 (26.0)	205 (27.9)	0.747
Professional and technical personnel	213 (11.2)	129 (11.0)	84 (11.4)	
Manufacturers	84 (4.4)	48 (4.1)	36 (4.9)	
Service personnel	559 (29.3)	346 (29.4)	213 (29.0)	
Clerical staff or students or others	222 (11.6)	140 (11.9)	82 (11.2)	
Unemployed	322 (16.9)	207 (17.6)	115 (15.6)	
Family annual income (RMB) thousands				
> 300	102 (5.3)	57 (3.0)	45 (6.1)	0.831
200∼300	157 (8.2)	97 (5.1)	60 (8.2)	
100∼200	644 (33.7)	399 (20.9)	245 (33.3)	
50∼100	704 (36.9)	433 (22.7)	272 (37.0)	
< 50	296 (15.5)	184 (9.6)	112 (15.2)	
*Missing data*	7 (0.4)	5 (0.3)	2 (0.3)	
Lifestyles				
Smoking status				
Never	857 (44.9)	518 (44.1)	339 (46.1)	0.071
Former	405 (21.2)	234 (19.9)	171 (23.3)	
Current	587 (30.7)	380 (32.3)	207 (28.2)	
*Missing Data*	61 (3.2)	43 (3.7)	18 (2.4)	
Alcohol drinking				
Almost never	489 (25.6)	297 (25.3)	192 (26.1)	0.432
Less than once a month	962 (50.4)	599 (51.0)	363 (49.4)	
Less than once a week	202 (10.6)	126 (10.7)	76 (10.3)	
More than once a week	196 (10.3)	110 (9.4)	86 (11.7)	
*Missing Data*	61 (3.2)	43 (3.7)	18 (2.4)	
Body mass Index (BMI) (kg/m^2^)				
< 18.5	39 (2.0)	29 (2.8)	10 (1.6)	0.183
18.5–23.9	748 (39.2)	457 (43.7)	291 (42.1)	
24-27.9	684 (35.8)	398 (38.0)	286 (41.5)	
≥ 28	265 (13.9)	163 (15.5)	102 (14.8)	
Missing Data *	174 (9.1)	128 (10.9)	46 (6.3)	
Doctor’s diagnosed disease				
Any disease				
No	1623 (85.0)	999 (85.0)	624 (84.9)	0.232
Yes	196 (10.3)	112 (9.5)	84 (11.4)	
*Missing Data*	91 (4.8)	64 (5.4)	27 (3.7)	
Mental Health ^‡^				
Depression (0–60)				
< 16	1253 (65.6)	779 (66.2)	474 (64.5)	0.661
≥ 16	485 (25.4)	296 (25.2)	189 (25.7)	
*Missing Data*	172 (9.0)	100 (8.5)	72 (9.8)	
Anxiety (20–80)				
< 50	1671 (87.5)	1035 (88.1)	636 (86.5)	0.607
≥ 50	63 (3.3)	37 (3.1)	26 (3.5)	
*Missing Data*	176 (9.2)	103 (8.8)	73 (9.9)	
Stress (0–40)				
0–13	597 (31.3)	378 (27.7)	219 (25.4)	0.634
14–19	667 (34.9)	405 (29.7)	262 (29.4)	
> 19	352 (18.4)	217 (30.7)	135 (32.4)	
*Missing Data*	294 (15.4)	175 (11.8)	119 (12.8)	
Having regular medication or surgeon treatment history				
No	1623 (85.0)	999 (85.0)	624 (84.9)	0.435
Yes	226 (11.8)	133 (11.3)	93 (12.7)	
*Missing Data*	61 (3.2)	43 (3.7)	18 (2.4)	
Sexual abstinence(day)				
< 2	4 (0.2)	3 (0.3)	1 (0.1)	0.825
2–7	1632 (95.7)	980 (83.4)	652 (88.7)	
> 7	69 (4.0)	41 (3.5)	28 (3.8)	
Missing *	205 (10.7)	151 (12.9)	54 (7.3)	
Sperm quality				
Sperm volume (ml)				
< 1.5	190 (9.9)	108 (9.2)	82 (11.2)	0.392
1.5-8	1470 (77.0)	893 (76.0)	577 (78.5)	
> 8	50 (2.6)	27 (2.3)	23 (3.1)	
Missing Data*	200 (10.5)	147 (12.5)	53 (7.2)	
Sperm concentration (ml×106)				
Azoospermia (= 0)	76 (4.0)	53 (4.5)	23 (3.1)	0.089
Oligozoospermic (1–14)	211 (11.0)	117 (10.0)	94 (12.8)	
Normal (≥ 15)	1391 (72.8)	838 (71.3)	556 (75.6)	
Missing Data*	232 (12.1)	168 (14.3)	64 (8.7)	
Sperm progressive motility				
Normal (≥ 32)	622 (37.1)	375 (31.9)	247 (33.6)	0.964
Abnormal (< 32)	1010 (60.2)	609 (51.8)	401 (54.6)	
Missing Data*	46 (2.7)	23 (2.0)	23 (3.1)	
Sperm morphology				
Abnormal(<4)	631 (33.0)	395 (62.6)	236 (37.4)	0.231
Normal (≥ 4)	729 (38.1)	433 (59.4)	296 (40.6)	
Missing Data*	552 (28.9)	348 (29.6)	204 (27.8)	

^†^ P-values in the Chi- square test are calculated based on available data, excluding missing data.

^‡^ Depression Scale, Self-Report Anxiety Report, and Pittsburgh Sleep Quality Scale. The validity and reliability of the Chinese version of the above questionnaires have been verified.

*There were significant differences in missing data between implantation failure and succeed group.

Table 2 displays the number, rate and crude odds ratio of implantation failure in paternal age groups. There were significant differences in implantation failure rate across six paternal age groups at 22-<25, 25-<30, 30-<35, 35-<40, 40-<45, and ≥ 45–57 years (*p* < 0.001), and the implantation failure increased with paternal age (trend *p* < 0.001). Taking the largest number of participants in the paternal age group of 30-<35 years as a reference group for analysis, we found that crude ORs of implantation failure in the age groups of 22-<25 and 25-<30 years were not significantly different from that the age group of 30-<35 years, but increased in the age groups of 35-<40 years (1.56, 95%CI 1.20–2.04), in 40-<45 years (2.07, 1.35–3.18) and in ≥ 45–57 years (4.07, 2.21–7.50) (Table 2).

**Table 2:** Number, % and OR of implantation failure by paternal age: AMCHS, China.

Paternal age	All participants	Implantation success	Implantation failure	P*	Univariate Logistic Regression Model		
(year)	*N* = 1910 (100%)	*n* = 1175 (61.5%)	*n* = 735 (38.5%)		OR	95% CI	P
22-<25	63 (3.3)	46 (73.0)	17 (27.0)	< 0.001	0.69	0.39–1.22	0.203
25-<30	637 (33.4)	410 (64.4)	227 (35.6)		1.03	0.83–1.29	0.789
30-<35	744 (39.0)	484 (65.1)	260 (34.9)		1.00		
35-<40	320 (16.8)	174 (54.4)	146 (45.6)		1.56	1.20–2.04	0.001
40-<45	95 (5.0)	45 (47.4)	50 (52.6)		2.07	1.35–3.18	< 0.001
≥ 45–57	51 (2.7)	16 (31.4)	35 (68.6)		4.07	2.21–7.50	< 0.001

* p value in Chi-square test < 0.001 and also linear-by-linear association *p* < 0.001.

Table 3 shows the adjusted odds ratio and 95%CIs of implantation failure in relation to advanced paternal age using different sets of adjustments for analysis. As there were no significant differences in the risk of IF among the age groups of 22-<25, 25-<30 and 30-<35 in all model analyses, we combined these groups into one group as a reference for analysis. Adjusting for urban-rurality living, annual family income, paternal smoking and alcohol consumption (Model 1), ORs of implantation failure in the age groups of 35-<40, 40-<45 and 45–57 compared to all in 22-<35 years were 1.53 (1.19–1.96), 2.04 (1.34–3.11) and 4.00 (2.17–7.38) respectively. Further adjustments for paternal depression, anxiety, history of any serious diseases, semen volume and morphology showed similar ORs to those in Model 1, except for reduction in the OR in the age group of 45–57 years older (adjusted OR 3.80, 2.50–7.07). Adding maternal age to the adjustment analysis (Model 3) made further reductions in the ORs in the age groups of 35-<40, 40-<45 and 45–57 years compared to those in Model 2; the corresponding ORs were 1.18 (0.89–1.57), 1.32 (0.81–2.16) and 2.24 (1.13–4.60) respectively, but the increased OR in the age group of 45–57 years remained significant. Additional adjustments for other factors from females (Model 4) showed similar ORs to those in Model 3.

**Table 3:** Adjusted odds ratio of implantation failure according to paternal age respectively: AMCHS, China.

Paternal age	Model 1			Model 2			Model 3			Model 4		
(year)	OR^1^	95% CI	P	OR^2^	95% CI	P	OR^3^	95% CI	P	OR^4^	95% CI	P
22-<35^†^	1.00			1.00			1.00			1.00		
35-<40	1.53	1.19–1.96	< 0.001	1.50	1.16–1.93	0.002	1.18	0.89–1.57	0.263	1.19	0.89–1.60	0.237
40-<45	2.04	1.34–3.11	< 0.001	2.06	1.34–3.16	< 0.001	1.32	0.81–2.16	0.272	1.31	0.79–2.16	0.296
45–57	4.00	2.17–7.38	< 0.001	3.80	2.50–7.07	< 0.001	2.24	1.13–4.6	0.021	2.13	1.06–4.29	0.034

^†^ due to no significant differences in odds of implantation failure among the groups of 22-<25, 25-<30 and 30-<35, the three groups were combined as one group as a reference group for analysis, increasing the statistical power in multiple adjusted regression analysis.

^1^ Model 1: adjusted for adjusted for urban-rurality, annual family income, male smoking and alcohol drinking,

^2^ Model 2: adjusted for factors in model 1, plus male depression (score), male anxiety, histories of any serious illness in men, semen volume, and semen morphology,

^3^ Model 3: adjusted for factors in model 2, plus female age (cont.),

^4^ Model 4: adjusted for factors in model 3, plus female occupation, female drinking alcohol, female BMI, female depression, female anxiety, infertility type, infertility duration, anovulation, and antral follicle count.

Separate data analysis by maternal age of 20-<30 years and ≥ 30 years (which was around a median of maternal age at 30.2 years in this study population of ART couples) showed a significant association of advanced paternal age with implantation failure in women aged ≥ 30 years, but not in younger maternal age groups. There were significant differences in the OR between the two groups (Table 4). Interaction effect analysis in the fully adjusted logistic regression model, also showed a significant interaction of advanced paternal age with maternal age on IF (Model 4 multiple adjusted OR 1.67, 95%CI 1.30–2.13, p = < 0.001, with paternal age ≥ 35 and maternal age ≥ 30). We further examined the risk of IF among men aged ≥ 35 years versus those aged 22-<35 years in each of three groups of maternal ages 20-<30, 30-<35 and 35–47 years, respectively (suppl Table 1). In the maternal age groups of both 30-<35 and 35–47 years, the increased risk of IF in men aged ≥ 35 years was significant, but not significant for the maternal age group of 20-<30, which further supported the interaction effect of advanced paternal age and maternal age on IF.

**Table 4:** Multiple adjusted ORs of implantation failure across different paternal age groups by maternal age and their differences for additive effect.

Paternal age (year)	Maternal age ≥ 30 years					Maternal age 20-<30 years					Differences in ORs	
	Participants, *n* = 1021		OR^†^	95% CI	p	Participants, *n* = 889		OR^†^	95% CI	p		
	All	IF (n, %)				All	IF (n, %)				ROR	P*
22-<35	593	222 (37.4)	1.00			851	282 (33.1)	1.00				
35–57^&1^	428	221 (51.6)	1.65	1.25–2.19	< 0.001	38	10 (26.3)	0.72	0.33–1.59	0.418	2.29	0.027

^†^ Model 4 adjustment.

^&1^ under maternal age ≥ 30 years, only one participate in the group of 40-<45 and one participant in the group of 45–57 (no event); thus these two groups were combined with the paternal age group of 35-<40 for analysis.

* one-side p value.

Separate data analysis by smoking status revealed that there was a significant association of advanced paternal age with implantation failure in men who smoked, but not in those who never-smoked (Table 5). The differences in the OR between these two groups approached conventional statistical significance (ROR 1.51, *p* = 0.084) (Table 5). In the multiple adjusted logistic regression model analysis, the interaction effect of advanced paternal age with smoking on IF was not significant (fully adjusted OR 1.19, 95%CI 0.82–1.73, *p* = 0.371, at paternal age ≥ 35 and smoking, data not shown in the tables). The three groups of paternal ages < 30, 30-<35 and ≥ 35 years showed borderline significant differences in the OR of implantation failure between the two groups, smoking and never-smoking, in the paternal age group ≥ 35 years (ROR 1.75, *p* = 0.071), following a non-significant increased ROR at the paternal age of 30-<35 years (1.17, *p* = 0.277). In addition, the duration of smoking cessation (in months) and total years of smoking were further analysed in Model 4 to examine their associations with IF risk. In the group of paternal age ≥ 35 years, each additional year of smoking was associated with 7.0% increased odds of IF (OR = 1.07, 95% CI: 1.01–1.14), whereas no association was observed in the younger paternal age group (OR = 0.99, 95% CI: 0.96–1.03). The difference in ORs between these two age groups was significant (ROR = 1.08, *p* = 0.014). However, the duration of smoking cessation (in months) was not significantly associated with IF in either age group, probably due to the smaller number of former smokers in the current AMCHS dataset, which could not provide enough study power in analysis for this.

**Table 5:** Multiple adjusted ORs of implantation failure across different paternal age groups by smoking status and their differences for additive effect.

Paternal age	Smoking					Never-smoking					Differences in ORs	
	Participants, *n* = 992		OR^†^	95% CI	p	Participants, *n* = 857		OR^†^	95% CI	p		
	All	IF (n, %)				All	IF (n, %)				ROR	P^‡^
22-<35	746	254 (34.0)	1.00			653	237 (36.3)	1.00				
35–57	246	124 (50.4)	1.50	1.02–2.21	0.040	204	102 (50.0)	0.99	0.64–1.55	0.967	1.51	0.084

^†^ Model 4 adjustment.

^‡^ one-side p value.

Separate data analysis examining alcohol consumption found a significant association of advanced paternal age with implantation failure among men who consumed alcohol consumption regularly, but no such association was observed in those who did not consume alcohol. There were significant differences in the association between the two groups of consuming alcohol and not consuming alcohol (Table 6). Interaction effect analysis in the multiple adjusted regression model also showed a highly significant interaction of advanced paternal age with alcohol consumption on IF (fully adjusted OR 10.71, 95%CI 2.92–39.34, p = < 0.001, in the paternal age ≥ 35 with alcohol consumption, data not shown in the table).

**Table 6:** Multiple adjusted ORs of implantation failure across different paternal age groups by alcohol drinking and their differences for additive effect.

Paternal age	Alcohol drinking					Non-Alcohol drinking						
	Participants, *n* = 1360		OR^†^	95% CI	p	Participants, *n* = 498		OR^†^	95% CI	p	Differences in ORs	
	All	IF (n, %)				All	IF (n, %)				ROR	P^‡^
22-<35	1024	343 (33.5)	1.00			375	148 (39.5)	1.00				
35–57	336	182 (54.2)	1.66	1.18–2.33	0.003	114	44 (38.6)	0.66	0.37–1.17	0.152	2.52	0.003

^†^ Model 4 adjustment.

^‡^ one-side p value.

## Discussion

Our cohort study examined (1) the association between paternal age and implantation failure; (2) the interaction effects of advanced paternal age with maternal age, paternal smoking and alcohol consumption on implantation failure. We found that the risk of IF started to increase with a paternal age ≥ 35 years and there appears to be a dose-responsive relationship, while at the paternal ages of 22–34 there was no significant increase in IF. The association of advanced paternal age (≥ 45 years) with IF was independent of other factors but was moderated by advanced maternal age reducing the association. It was significantly pronounced at a maternal age above 30 years. Our study also demonstrated that while advanced paternal age was correlated with a heightened risk of IF among male smokers, this pattern did not extend to never-smokers. There was also a strong interactive effect between advanced paternal age and alcohol consumption on the risk of IF.

Previous studies showed inconsistent findings in the association between advanced paternal age and IF. For instance, Kaarouch et al.^22^ recruited 83 couples with female partners aged ≤ 39 undergoing IVF treatment in France and found no significant differences in IF between younger (< 40 years) and older (≥ 40 years) paternal age groups. The findings of their study were limited by the small sample size and lack of adjustment for additional factors. In the USA, conducting a retrospective cohort study of 2063 couples at a single academic reproductive medicine centre from 2012 to 2018, Kasman et al. (2021)^10^ also reported no significant effect of advanced paternal age on IF. In contrast, other studies showed significant association of advanced paternal age with IF. de La Rochebrochard et al. (2006)^7^ extracted data from 59 centres in the French National IVF Registry to examine 1938 couples with bilateral tubal obstruction and found that the adjusted odds ratio of failure to conceive was 1.52 (95% CI 1.08–2.14) at a paternal age 30–34 years, 1.32 (0.92–1.89) at 35–39 years, and 1.70 (1.14–2.52) at paternal age ≥ 40 years versus < 30 years. Coban et al. (2022) conducted a retrospective cohort study including 4930 fresh oocyte donation cycles from 3995 couples who had fresh embryo transfers at British Cyprus IVF Hospital from April 2005 to February 2020 and found that pregnancy rates (as defined by β-HCG) were significantly different among the paternal age groups of ≤ 29 years old (81%), 30–34 (71.8%), 35–39 (68.3%), 40–44 (71%), 45–49 (60.7%) and ≥ 50 (61.4%)^23^ These conflicting findings possibly stem from inadequate adjustment analysis for confounding factors in some studies^24^, particularly the absence of adjustment for maternal age and lifestyle factors (such as paternal smoking and alcohol consumption)^21,22,25^. Omitting maternal age from analyses exploring the impact of paternal age on ART outcomes, leads to analytical problems due to the high correlation between paternal and maternal ages, raising concerns about collinearity. In our analysis, maternal age was considered after adjustment for all paternal factors (Model 3) to determine the extent to which maternal age mediated the association between paternal age and IF. Even after including maternal age in our adjustments, the positive association of paternal age with IF persisted, albeit with reduced magnitude. Our study’s comprehensive adjustments for confounding variables, including maternal mental health, infertility specifics and biological aspects of both males and females, established a significant positive relationship between paternal age and IF.

Previous studies in western countries showed that paternal age of around ≥ 40 years would significantly increase the risk of IF in ART settings. Our AMCHS study found that advanced paternal age could be associated with increased IF from ≥ 35 years, which was younger than those in western countries^7,26–28^, potentially indicating population-specific cut-offs that may differ due to racial or environmental factors. The significant interaction effect between paternal and maternal ages on IF could be attributed to a compromised oocyte repair capacity. During embryogenesis, oocytes can repair sperm-derived DNA damage. However, advanced paternal age increases the extent of such damage, which may not be fully compensated for when maternal age exceeds 30^29^. The evidence from our study underscores the importance of considering paternal age in family planning. Future research should aim to unravel the mechanisms underlying paternal and maternal age thresholds, which may vary by race and environmental exposure. There is established evidence that paternal smoking increases the risk of implantation failure^14,30^ Tobacco smoke constituents directly interact with spermatozoa, potentially leading to DNA damage, which in turn may precipitate implantation failure^31,32^. Our AMCHS corroborates this by demonstrating a significant elevation in the risk of IF associated with advanced paternal age in individuals who were current and former smokers, compared to never-smokers. This suggests that paternal smoking may exacerbate the adverse effect of advancing paternal age on IF among couples undergoing ART treatments. Moreover, our data demonstrated a possible cumulative effect of tobacco exposure in the advanced paternal age group. It is possible that prolonged smoking with advanced paternal age could compound sperm DNA damage and oxidative stress over time^33^. More studies are needed to elucidate the different impacts of smoking on sperm quality in paternal age < 35 and ≥ 35 years, considering both genetic and epigenetic factors.

Rossi et al. suggested that paternal alcohol consumption increases the risk of IF (OR 1.36, 95%CI 1.04–1.78)^34^. Our research identified a substantial interaction between advanced paternal age and paternal alcohol consumption on IF. This is in line with previous studies^34,35^ indicating the detrimental effects of excessive alcohol consumption on semen quality and its consequent association with implantation failure. For example, conducting a prospective cohort study of 965 male patients Borges et al.^36^ found that alcohol consumption significantly influenced sperm count/ml (*B*: −12.527, slope: 42.255, *p* = 0.040) and sperm DNA fragmentation (*B*: 5.833, slope: 9.680, *p* = 0.002), which may lead to IF^32^. Therefore, it was not surprising that we observed the interactive effect between advanced paternal age and alcohol consumption on IF. The additive effect observed in our study, where advanced paternal age significantly intensified the risk of IF among regular alcohol consumers, but not among abstainers, underscores the importance of avoiding alcohol for men aged ≥ 35 years in ART settings to enhance implantation success. It is imperative that paternal smoking and alcohol consumption are considered critical covariates when evaluating the impact of advanced paternal age on male infertility and ART outcomes.

### Strengths and limitations of the study

The main contribution of this study, beyond the intrinsic importance of studying the impact of advanced paternal age on IF, lies in what it tells us about the paternal age cut-off point increasing the risk of IF with a maternal age contribution, factors influencing this association and its interactive effects with maternal age, paternal smoking and alcohol consumption. As far as we know, our study is the first to report differences in the impact of advanced paternal age on IF between smokers and never-smokers, and between men who consumed alcohol and those who did not, addressing that men with adverse lifestyles had a greater risk of IF and worsening the impact of advanced paternal age on IF. Our study included important co-variables for adjustment such as female age, reasons for infertility, male sperm quality and couples’ socioeconomic status, thus the confounding effect is minimised. Different sets of confounding adjustments would allow us to identify factors influencing the association. Furthermore, in our study infertile couples were consecutively recruited and a completed baseline questionnaire before the first FET to avoid selection bias. The study has limitations. First, all participants in the cohort were from the areas of Hefei, Anhui, China, and caution should be exercised when generalizing the findings to all infertile couples across the whole of China who received ART treatment. Second, the number of participants in the current cohort study was not large so that when we examined the interactive effects of paternal age with maternal age, paternal smoking and alcohol consumption, the 95%CIs were wide. In the future, large cohort studies are required to examine the interactions between different paternal age levels and these confounding factors.

## Conclusion

In the context of ART in China, our study discovered that paternal age ≥ 35 years is correlated with an augmented risk of IF. Notably, this risk is compounded when both partners are of advanced age. Paternal smoking and alcohol consumption exacerbate the risk of IF alongside advancing paternal age. These findings underscore the necessity of emphasizing the implications of delayed childbearing in public health communications, as such delays significantly contribute to heightened IF risks among ART patients. Smoking cessation and the avoidance of alcohol by prospective fathers aged ≥ 35 years can mitigate the adverse effects of increased paternal age on IF, thereby improving the prospects of implantation success in ART treatments.

## Electronic supplementary material

Below is the link to the electronic supplementary material.

Supplementary Material 1

## Data Availability

All data supporting the findings of this study are available within the paper and its Supplementary Information. Potential collaborators are invited to contact the corresponding author (caoyunxia5972@ahmu.edu.cn).
