# Supplementary material for: Effects of paternal age and interaction with smoking, alcohol consumption and maternal age on implantation failure in women undergoing ART
Source: Sci Rep. 2025 Jul 2;15:23452. doi: 10.1038/s41598-025-03651-y (PMC12222860; doi:10.1038/s41598-025-03651-y)
Supplement: Supplementary file 1 — Supplementary Material 1 [file 41598_2025_3651_MOESM1_ESM.docx]

**Suppl Table 1. Adjusted ORs of implantation failure in paternal age of ≥ 35 years versus <35 years by different maternal age groups**

| **Maternal age** |  | **Paternal age** | | | | |  | **Differences in IF between two groups of paternal age** | | | | |
| --- | --- | --- | --- | --- | --- | --- | --- | --- | --- | --- | --- | --- |
| **(years)** |  | ≥35 years | |  | 22-<35years | |  | OR^†^ | 95%CI | | p |  |
|  |  | All | Implantation failure, n(%) |  | All | Implantation failure, n(%) |  |  |  |  |  |  |
|  |  |  |  |  |  |  |  |  |  |  |  |  |
| 20-<30 ^&1^ |  | 38 | 10 (26.3) |  | 851 | 282 (33.1) |  | 0.72 | 0.33 | 1.59 | 0.418 |  |
| 30-<35 |  | 167 | 78 (46.7) |  | 536 | 203 (37.9) |  | 1.49 | 1.02 | 2.19 | 0.041 |  |
| 35-47 ^&2^ |  | 261 | 143 (54.8) |  | 57 | 19 (33.3) |  | 3.22 | 1.50 | 6.91 | 0.003 |  |

^†^ Model 3 adjustment

^&1^ Combined groups due to a small number of in women aged 20-<25 years under the paternal age of ≥35 years (0 events in 2 participants)

^&2^ Combined groups due to a small number of in women aged 40-47 years under the paternal age of <35 years (2 events in 5 participants)
